# Higher Plant-Derived Biostimulants: Mechanisms of Action and Their Role in Mitigating Plant Abiotic Stress

**DOI:** 10.3390/antiox13030318

**Published:** 2024-03-06

**Authors:** Sara Esperanza Martínez-Lorente, José Manuel Martí-Guillén, María Ángeles Pedreño, Lorena Almagro, Ana Belén Sabater-Jara

**Affiliations:** Department of Plant Biology, Faculty of Biology, Campus of Espinardo, University of Murcia, 30100 Murcia, Spain; sesperanza.martinez@um.es (S.E.M.-L.); josemanuel.martig@um.es (J.M.M.-G.); mpedreno@um.es (M.Á.P.); lorena.almagro@um.es (L.A.)

**Keywords:** agriculture, climate change, abiotic stress, reactive chemical species, plant biostimulants, higher plant-derived biostimulants, mechanisms of action

## Abstract

Modern agriculture is being challenged by deteriorating edaphoclimatic conditions and increasing anthropogenic pressure. This necessitates the development of innovative crop production systems that can sustainably meet the demands of a growing world population while minimizing the environmental impact. The use of plant biostimulants is gaining ground as a safe and ecologically sound approach to improving crop yields. In this review, biostimulants obtained from different higher plant sources are presented under the term higher plant-derived biostimulants (hPDBs). Their mechanisms of action regulate physiological processes in plants from germination to fructification, conditioned by responses induced in plant mineral nutrition and primary metabolism, specialized metabolism, photosynthetic processes, oxidative metabolism, and signaling-related processes. The aim of this review is to collect and unify the abundant information dispersed in the literature on the effects of these biostimulants, focusing on crops subjected to abiotic stress conditions and the underlying mechanisms of action.

## 1. Introduction

One of the main challenges facing modern agriculture is finding a sustainable way of feeding the growing world population, which the United Nations estimates will increase by nearly 2 billion people over the next 30 years, considering the decrease in crop area [1]. The use of agrochemicals to boost food production is becoming increasingly unsustainable due to indiscriminate use, prompting stricter regulations. Moreover, there is growing consumer demand for ecological products, particularly since the COVID-19 pandemic. Products with “BIO” or “ECO” labels, indicating sustainable production systems free of agrochemicals, are regarded as healthier alternatives to conventionally produced foods [2]. The need to maintain product quality standards at the highest level is evidenced by the data provided by the Food and Agriculture Organization of the United Nations (FAO), which highlights that 90% of vitamin C and 60% of vitamin A consumed by the human population come from agricultural crops [3]. Therefore, it is important to guarantee both quality standards and food security of the population.

In the move toward sustainable agriculture, the Farm to Fork Strategy, included in the European Green Deal, has set a deadline of 2030 to reduce the use of chemical pesticides by 50% as well as soil nutrient losses by at least 50%, which in turn should cut the use of fertilizers by at least 20%, with the intention of allocating 25% of agricultural areas to organic farming [4,5].

These limitations and reductions in the use of agrochemicals have been the consequence of years of their abuse in agriculture, facts that have manifested themselves in the deterioration of soils, along with the potential damage to human health and the environment. Among them, the uncontrolled use of nitrogen-rich fertilizers has triggered eutrophication processes, with consequent severe environmental damage, as well as nitrate accumulation in plants, exceeding the regulation limits that allow their consumption. In fact, the nitrates present in these foods are metabolized into potentially carcinogenic compounds that are harmful to human health [6].

Another challenge facing modern agriculture is the diminishing availability of land for crop production due to climate change. Alterations in rainfall patterns and escalating global temperatures are leading to the aridification of arable lands, rendering them unprofitable for food production [7].

All these factors indicate there is an urgent need to develop more sustainable agricultural systems capable of providing for the growing population while minimizing the environmental impact. Overcoming the increasingly adverse anthropological and edaphoclimatic conditions that limit production performance is imperative. Moreover, the long processes of genetic improvement through breeding developed over the years in crops are reaching the limit of their potential. Given that improving crop tolerance to climate change by genetic modifications or in vitro selection takes years to accomplish, it is of paramount interest to search for alternative strategies with a more immediate impact [3].

## 2. Climate Change and Plant Abiotic Stress

**Figure 1:**
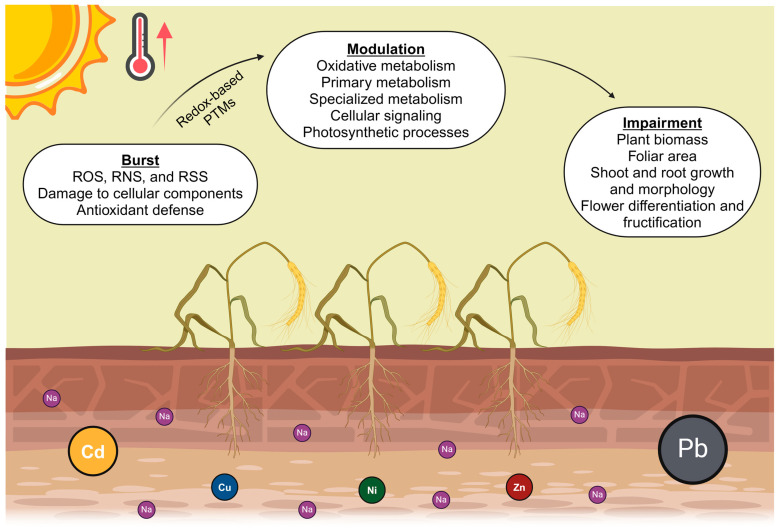
Schematic representation of the main abiotic stresses (drought, salinity, heat, and PHEs) mentioned in the text and the responses triggered in plants subjected to them. Created with BioRender.com, accessed on 1 March 2024.

Climate change, in essence, is a phenomenon that affects the entire planet at the global level. Its manifestation includes variations in weather conditions, especially the increase in global temperatures and the disruption of precipitation patterns, with serious consequences for global agricultural production. It results in a reduction in annual yields in terms of both quantity and quality, as well as the annual economic performance of the crops [8]. The adverse conditions caused by climate change result in abiotic stresses on plants. Among these stresses, extreme temperatures, prolonged water deficit, high soil salinity, and contamination by potentially hazardous elements (PHEs) stand out (Figure 1).

Stress due to extreme temperatures occurs when the temperature range for plant growth and development is exceeded above or below the basal maximum and minimum. Extreme cold and sudden drops in temperature, as well as extreme heat, which occurs more frequently, restrict and limit the physiological processes of temperature-sensitive plants, affecting their growth and development [9]. Stress due to water deficit or drought occurs when the availability of water in the soil decreases drastically, generating a water imbalance in the transpiration rate, which exceeds the intake of water absorbed by the root system, or causing the plants to close their stomata to avoid the previous, and thus preventing them from absorbing CO_2_ for photosynthesis. As well as insufficient precipitation, water deficit or drought are caused by increased water evaporation under conditions of prolonged heat, leading to elevated soil salt concentrations that impede optimal plant growth [9,10]. Similar effects are caused by soil contamination with PHEs, such as boron (B), aluminum (Al), copper (Cu), zinc (Zn), lead (Pb), vanadium (V), lanthanum (La), nickel (Ni), arsenic (As), cadmium (Cd), and chromium (Cr), essentially arising from human activities near crop areas, such as industrial effluents and global pollution [11].

As sessile organisms, plants are exposed throughout their life cycle to different abiotic stresses, which can act individually, but generally occur in combination. This aggravates the damage and generates unique responses that cannot be directly extrapolated from the responses to the individually applied stresses, involving metabolic and physiological mechanisms that allow them to acclimate and, consequently, tolerate exposure to adverse conditions [12,13]. The immediate response of plants to stress is a massive increase in the production of reactive chemical species, including reactive oxygen (ROS), nitrogen (RNS), and sulfur (RSS) species. These molecules, present at low concentrations in plants under non-stress conditions, act as signaling molecules in cellular events, but under stress conditions, their overproduction disrupts redox homeostasis and damages essential biomolecules and cellular components. This oxidative, nitrosative, or nitro-oxidative stress can potentially lead to cell death. However, cells employ diverse mechanisms to ameliorate stress, achieving tolerance and acclimation, allowing plant survival. These mechanisms include the expression of genes related to antioxidant defense; the synthesis and activation of enzymes and antioxidant compounds (e.g., melatonin, glutathione, ascorbic acid, and flavonoids) that counteract the overproduction of reactive chemical species; and the transcription of genes encoding heat shock proteins, LEA proteins, osmoprotectant biosynthesizing enzymes, stress-related transcription factors, aquaporins, and ion channels, among others [14,15,16,17].

In turn, ROS, RNS, and RSS have the capacity to generate post-translational modifications (PTMs) in target proteins, altering their physiological, structural, and functional properties, which affects their stability and affinity with other proteins, biomolecules, or related metabolites. The majority of PTMs are highly specific of each protein, affecting particular amino acid residues with physical–chemical properties that render them susceptible to these types of redox-based PTMs. The modifications induce conformational changes that can either promote or inhibit enzyme catalytic activity. After reactive chemical species burst, the proteome susceptible to these redox-based PTMs acquires new modifications that can compromise structural proteins and key enzymes, but also initiate the plant acclimation processes. It has been described that reactive species initiate plant acclimation processes by modifying key amino acid residues in proteins associated with oxidative metabolism, primary and specialized metabolisms, cellular signaling, photosynthetic processes, photorespiration, and Calvin–Benson cycle [18]. The modification of structural proteins and key enzymes of cellular metabolism determines the correct functioning of essential physiological processes such as cell division, photosynthesis, chlorophyll biosynthesis, photorespiration, balance between water absorption and transpiration, stomatal opening and closing, and translocation of essential nutrients [9,18].

Consequently, the imbalance of plant homeostasis induces morphological changes, such as reduced growth of aerial parts, including leaf expansion, thickness, and area; growth and deepening of roots in search of water; and disruption of the differentiation of male and female flowers. Ultimately, these changes negatively affect the total biomass, plant vigor, and fructification [19,20,21].

## 3. Plant Biostimulants: An Emerging Ecological Alternative

The use of biostimulants among sustainable agricultural practices is gaining ground as a promising, safe, and ecological alternative to improving crop production performance.

Prior to the term “biostimulant”, the terms “biogenic stimulators” or “biogenic stimulants” were used to refer to substances synthesized in tissues under stressful, but not lethal, conditions, which stimulated the vital reactions of the organism [22]. The term “biostimulant” was used for the first time in a research article by Russo and Berlyn published in 1991 [23]. These authors went on to define biostimulants in 1992 [24] as “non-nutritional products that may reduce fertilizer use and increase yield and resistance to water and temperature stresses”, also remarking that they “stimulate plant growth [when used] in relatively small amounts” [25].

Despite the recent exponential increase in research on biostimulants, controversy remained regarding their precise definition. Until official regulation by the European Union in 2019, the status of biostimulants differed between member states and they were marketed as biofertilizers in mixtures with nutritional elements [26,27]. Nevertheless, by 2019, a consensus on the key attributes of a biostimulant had been reached. These include the ability to act on plant homeostasis at low doses, improve plant growth, induce a more efficient use of nutrients and water, modulate abiotic stress response, and exert synergistic effects resulting from the combination of bioactive compounds [25]. Finally, Europe laid the foundations for the regulation of these products, with the publication of Regulation (EU) 2019/1009 of the European Parliament and of the Council of 5 June 2019, where biostimulants are defined as [28]:

“A plant biostimulant shall be an EU fertilising product the function of which is to stimulate plant nutrition processes independently of the product’s nutrient content with the sole aim of improving one or more of the following characteristics of the plant or the plant rhizosphere:

(a) nutrient use efficiency,
(b) tolerance to abiotic stress,
(c) quality traits, or
(d) availability of confined nutrients in the soil or rhizosphere.”

Accordingly, it was established that the main difference between a biofertilizer and a biostimulant is that a biofertilizer participates in the provision of nutrients to the plant, whereas biostimulants favor nutrient acquisition [29]. Furthermore, in contrast to plant defense elicitors, which provide a protection against biotic stress by inducing systemic acquired resistance, biostimulants act by providing tolerance to abiotic stress [30].

The lack of a clear definition due to the appearance of the concept of biostimulants in the scientific world has compromised the way of properly classifying them. The wide diversity of biostimulants, in both origin and composition, as well as the physiological functions triggered by their application, has complicated their classification, although the scientific community has widely accepted the proposal of Patrick du Jardin in 2015 [31], who divided biostimulants into seven categories. Humic substances, which include humic and fulvic acids, correspond to heterogeneous organic molecules resulting from the decomposition of organic remains in the soil. Protein hydrolysates (PHs) are mixtures of variable proportions of free amino acids, oligopeptides, and polypeptides, obtained through the chemical and enzymatic hydrolysis of proteins from various sources, both animal and plant, generally obtained from industrial by-products and waste. Seaweed and botanical extracts are variable and heterogeneous mixtures that, depending on the origin of the extract, include complex polysaccharides, phenolic compounds, and hormones, among others. Biopolymers, which include a wide diversity of molecules of highly variable size and characteristics, are obtained by extraction or industrial synthesis. Inorganic compounds correspond to beneficial elements, mainly Al, cobalt (Co), Na, selenium (Se), and silicon (Si), present as different inorganic salts. Beneficial fungi and bacteria constitute a very broad and varied group that include symbiotic microorganisms and plant growth promoters [31].

**Figure 2:**
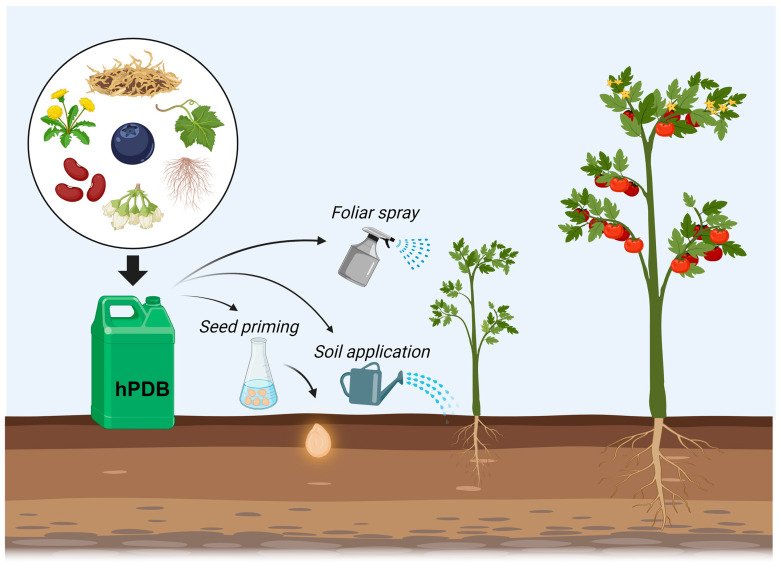
hPDB main sources and application method. Created with BioRender.com, accessed on 31 January 2024.

Despite their heterogeneity, biostimulants are generally applied in three different ways: through seed priming, application to soil (or to nutrient solution for hydroponic crops), and in foliar spray (Figure 2). In seed priming, the seeds are soaked in a solution containing the biostimulant to enable ingredient penetration. In the case of soil application, the product effectiveness depends on adequate soaking, while the application in hydroponic cultivation is more straightforward, with the desired concentration simply being added to the nutrient solution. In foliar spraying, the biostimulant is applied in solution when the seedling reaches a minimum age, typically after the generation of several true leaves. The frequency of application varies according to the biostimulant used [32]. The efficacy of a biostimulant hinges on the penetration of active ingredients into the seeds, roots, or leaves, and their assimilation is based on factors such as molecular structure, particle size, and solubility. Additives are commonly used to optimize solubility, absorption capacity, and penetration into the plant material [33].

Biostimulants from natural sources have attracted attention for their sustainability, ecological advantages, and biodegradability, ensuring minimum or null environmental impact. Among them, higher plant-derived biostimulants (hPDBs) stand out, above animal-derived biostimulants (ADBs), because their sustainability and profitability are superior. In fact, the production of ABDs generates more CO_2_ emissions (+57%) and consumes more energy (+26%) and water [34]. Furthermore, animal-derived by-products, the source of most ADBs, represent a potential risk for the consumer through disease transmission [35]. Regarding microorganism-derived biostimulants, which have also attracted a lot of attention, there is controversy about their application, because some microbial products are not normally present in agricultural fields [22].

We define the term hPDBs as group of biostimulants that gather PHs, extracts from plant by-products, whole plants or specific organs, purified metabolites, and cell cultures derived from higher plants, included in the Tracheophyta phylum (Figure 2). Biostimulants, by definition, are effective in inducing physiological processes in plants that improve their growth and acclimation to stress, concluding in an increase in crop yield. Furthermore, the generation of abundant plant by-products after obtaining the marketable and consumable parts of the crop is very common, so they can be revalued and used to increase crop production and profitability while reducing the environmental impact of waste disposal [36]. hPDBs are of great interest due to the enormous variability of biomolecules that compose them, from peptides to specialized metabolites, which is the reason they can potentially induce a multitude of physiological processes that lead to an increased crop yield [32].

Therefore, this review brings together the knowledge, abundant but widely dispersed in the literature, concerning hPDBs and their mechanisms of action when they are applied to plants, focusing on crops subjected to abiotic stress conditions. Table 1 shows the main hPDBs used in different crops reviewed, as well as their application method.

## 4. Higher Plant-Derived Biostimulants: Mechanisms of Action

Firstly, it is necessary to highlight the differences between mechanisms and modes of action. According to Yakhin et al. (2017) [22], the mode of action refers to the “specific effect on a discrete biochemical or regulatory process”, while the mechanism of action involves the “impacts on general biochemical or molecular pathways or physiological processes”. As biostimulants have not been characterized at the very specific level necessary to establish their modes of action, their functions in plants are mainly understood based on the underlying mechanisms of action. The research developed on the mechanism of action of biostimulants requires elucidating the composition and bioactive ingredients of the formulation, as well as the mechanism of action that induces modifications in plant metabolism. To perform these determinations, powerful omics tools (transcriptomics, metabolomics, proteomics, and phenomics) can generate abundant data on changes in mRNA transcripts, metabolites, proteins, and phenotypes. Thus, the application of these techniques is essential to be able to obtain this knowledge, crucial to customize formulations for specific crop applications [35,129,130]. Furthermore, determining the effect of biostimulants is not only recommended to obtain an optimal formulation, but members of the European Biostimulant Industry Council (EBIC) consider it an essential requirement for their commercialization [131].

The wide range of plant processes in plants modulated by biostimulants are presented here in two sections, beginning with the mechanisms of action at the molecular or cellular level and then in terms of the whole plant.

### 4.1. Cellular and Molecular Levels

#### 4.1.1. Plant Mineral Nutrition and Primary Metabolism

Nutrient use efficiency and assimilation, as well as carbon (C) energy metabolism, are processes of plant primary metabolism, which constitutes a large part of the physiological reactions the plant carries out to perform its vital functions. The uptake and assimilation of nutrients from the soil play a decisive role in proper plant development, as nutrients are needed to produce essential metabolites and enzymes in addition to acting as cofactors. Various types of biostimulants are reported to enhance the uptake of phosphorus (P), sulfur (S), calcium (Ca), magnesium (Mg), potassium (K), iron (Fe), and Zn, increasing their concentration and intracellular bioavailability in spinach [105,106], perennial wall rocket [98,99], rocket [103], tomato [117,122], maize [83], olive tree [93], kinnow mandarin [66], cannabis [42], and rose-scented geranium [104]. In a study carried out in tomato plants treated with an alfalfa-based PH, an increase was observed in the transcription of transporters related to ion uptake, such as sulfate transporters *SULTR 2;1* and *SULTR 3;1*, Cu transporters, phosphate transporter *PT2*, Fe-phytosiderophore transporter protein yellow stripe 1 (*YS1*), K channels, and ABC transporters [123]. Thus, by improving fertilizer efficiency, biostimulants could reduce its application dosage. Additionally, hPDBs exhibit protective effects against the excessive accumulation of PHEs in plants grown in contaminated soils, modulating the levels of Cu [55], Cd [46], Pb, and Ni [111]. The application of biostimulants has also proven beneficial in mitigating the toxic effects of salt stress. An imbalance in the K/Na ratio causes ionic and osmotic stress in the cell, which also affects the correct assimilation of other ions. This imbalance was alleviated by biostimulants in maize [84,132], camelina [41], common bean [45,49], pea [97], lettuce [73], eggplant [54], and okra [92]. Moreover, this approach was also able to balance the levels of other essential ions such as Ca, S, P, K, Mg, and (Mn) [45,53,69,70].

Nitrogen (N) has special relevance among the nutrients assimilated by plants, as it is used to form amino acids after fixation as organic N from nitrate. The application of hPDBs has resulted in a higher N content in leaves of different crops, including cascading geranium [43], spinach [67], maize [72,83], dwarf pea [79], tomato [122], and rocket [103], an effect also observed under salt stress conditions [45,50,53,74]. This increase in N concentration allows for a reduction in fertilizer dosage, thus maintaining nitrate levels within the legal limits set by the European Commission [6,71]. Recent studies in tomato plants treated with different PHs found that greater N assimilation was not only related to the enhanced transcription of genes encoding transporters of nitrate (*NTR2*) and ammonium (*AMT1.1* and *AMT1.2*), increasing the translocation from soil to the root, but also to an upregulation of the genes involved in N metabolism and the synthesis of amino acids such as nitrate reductase (*NR*), nitrite reductase (*NiR*), aspartate aminotransferase (*AST*), glutamine-dependent asparagine synthetase (*ASN1*), ferredoxin-dependent glutamate synthase (*GLT*), NADH-dependent glutamate synthase (*GLS*), glutamate dehydrogenase (*GDH*), and glutamine synthetase (*GS1* and *GS2*) [115,123]. Moreover, an increase in the activity of NR, GS, and glutamate synthase (GOGAT) [44] was demonstrated in maize [85,86]. In fact, in maize plants subjected to saline conditions, treatment with an alfalfa-based PH also increased the activity of GS and GOGAT [87]. Therefore, an enhanced uptake of N and its consequent incorporation into amino acids also improves its bioavailability in plant tissues [52,105]. Furthermore, transcriptomic experiments have revealed that tomato plants treated with hPDBs have higher transcript levels of the amino acid transporter *AAT1* [115], organic cation/carnitine transporters, nodulin *MtN21*, glutathione-conjugate transporter *MRP4,* and other N-associated enzymes (aspartyl protease, glutamate dehydrogenase, and serine decarboxylase), as well as genes encoding proteins involved in protein synthesis and modification (aminoacyl-tRNA synthetases, elongation factors Tu and 1-alpha, translation initiation factors, and ubiquitin-conjugating enzymes) [123]. This surge in protein synthesis leads to an increase in total proteins, a parameter improvement described in maize [83], wheat, rice, mung bean, and common bean plants [44,91] following biostimulant application. Under stress conditions, higher protein [94,124] and amino acid [84] concentrations were reported in treated versus untreated plants, with a notable increase in the amount of proline, an amino acid involved in stress responses [50,74]. In grapevines grown under conditions of water scarcity, the application of commercial biostimulant formulations Trainer^®^ and Stimtide^®^ increased the accumulation of bifunctional aspartokinase/homoserine dehydrogenase 2, involved in protein metabolism [61].

Sugars and carbohydrates are essential for plant growth and development, serving as both a source of energy and essential components for the synthesis of structural molecules. The application of biostimulants can increase the levels of soluble sugars in plants, as has been shown in maize [81], kinnow mandarin [66], rocket [103], habanero pepper [65], and tomato [117], among others. Furthermore, in tomato plants, biostimulants enhanced the transcription of genes encoding enzymes involved in sugar metabolism (polygalacturonase, pectinesterase, starch synthase, sucrose synthase, cellulose synthase, and inositol oxygenase), as well as key enzymes of C metabolism (such as fumarate dehydrogenase, malate dehydrogenase, phosphoenolpyruvate carboxylase, and phosphoenolpyruvate carboxylase kinase 2) [123]. In monocotyledons, such as maize, the application of hPDBs resulted in increased transcription and activity of malate dehydrogenase, isocitrate dehydrogenase, and citrate synthase [85]. A striking response was observed in maize plants treated with cellulosolitic dry apple hydrolysate or a dry blueberry cool extract, which led to an increase in fructose concentration and a decrease in glucose and sucrose. By stimulating plant respiration, the treatment promoted the consumption of glucose, thus limiting its availability for sucrose synthesis and resulting in the accumulation of fructose [83].

Sugars and carbohydrates, in turn, are used for the synthesis of other primary metabolites, such as nucleosides, in whose chemical structure a ribose or deoxyribose pentose is attached, together with a nitrogenous base, constituting the different types of nucleosides. It has been shown that treatment with different hPDBs increases thymidine accumulation in habanero pepper fruits [65].

The application of hPDBs in crops subjected to stress conditions generally causes an increase in soluble sugar concentration, providing osmoprotective benefits in both dicotyledons [48,111] and monocotyledons [84]. In Arabidopsis plants exposed to salinity and treated with plant-derived PHs, maltose accumulation was observed. Maltose, a product of starch degradation [37], plays a protective role in response to stress by safeguarding the electron transport chain of the chloroplast, thereby preserving photosynthetic function [133]. The higher maltose levels could be related to the increased activity of α-amylase, which degrades starch to maltose units, as observed in camelina [41] and wheat [124] treated with sorghum water extract under salinity conditions. Furthermore, the application of narrow-leaf cattail extracts in pea plants subjected to salinity preserved metabolic activity, quantified as the amount of formazan accumulated in the viability test, and the respiration rate was higher compared to untreated plants [95].

With respect to lipid metabolism, an increase in the transcription of genes encoding lipases has been described in tomato plants treated with an alfalfa-based PH [123], whereas other studies in tomato reported an accumulation of structural lipids such as hexadecane-diol and hydroxy stearate [112]. Under limited water availability, the pretreatment of tomato plants with Trainer^®^ caused a modulation, both positive and negative, of membrane lipids and sterol accumulation [118].

#### 4.1.2. Specialized Metabolism

Specialized metabolites, distinct from compounds essential for plant growth, development, and reproduction, are often restricted to a particular taxonomic group. They are produced from primary metabolites, normally at specific stages of development and in response to certain conditions. These compounds intervene in the dynamic interactions between plants and their environment, acting as structural or regulatory molecules or hormones. Specialized metabolites exhibit diverse ecological functions, serving as attractants, repellents, or even natural pesticides. A large proportion of specialized metabolites are pigments responsible for the colors of flowers and fruits. These pigments are essential for reproduction, attracting species crucial for pollination, and seed dispersal. Specialized metabolites also participate in protective functions under both abiotic and biotic stress conditions. Commonly, most of these compounds participate in responses to both types of stresses. Thus, the modulation in the content of these metabolites by hPDBs would not only determine their tolerance to abiotic stress, but also suggest a potential role as plant defense elicitors against biotic stress. Based on their chemical structure, specialized metabolites can be classified into three groups: terpenoids, phenolics, and nitrogen-containing compounds [134,135,136,137].

*Terpenoids*

Terpenoids are synthesized from acetyl-CoA, which is transformed via the mevalonic acid (MVA) and 2-C-methylerythritol 4-phosphate (MEP) pathways into the isoprene unit isopentenyl pyrophosphate (IPPP), the main terpene building block [136]. hPDBs exhibit a dual regulatory role in terpenoid accumulation. They are reported to negatively modulate terpenoid levels under various conditions, including those of control conditions [37,112], water scarcity [118], and saline stress [68], or increase their concentration under both control conditions [112,116] and Cu toxicity [82]. In tomato, the application of an alfalfa-based PH led to an upregulation of terpene biosynthetic genes, including hydroxy-3-methylglutaryl coenzyme A reductase, isoflavone reductase, and terpene synthase [123].

Carotenoids, a subset of terpenoids that act as antioxidants and pigments involved in photosynthesis, are positively influenced by various hPDBs. Biostimulant application enhanced carotenoid accumulation in leaves of different plant species, both under control [6,103,104] and stress conditions [95], and increased lycopene concentration in tomato fruits [113,114,117].

Terpenoid phytoalexins, which function as defense compounds, were found to accumulate after treatment with biostimulants [112,116]. The accumulation of phytoalexin precursors and terpenoid phytoalexins in lettuce was especially remarkable under abiotic stress conditions [69]. On the other hand, in Genovese basil, treatment with Trainer^®^ alleviated terpenoid and phytoalexin accumulation after exposure to high Cu levels, which indicates the formulation exerted a protective effect against the oxidative stress induced by this metal [55].

Terpenoids are also among the volatile compounds responsible for the quality of certain crops. In different types of grapevine, the fruits had higher terpene and norisoprenoid concentrations after treatment with vine-shoot extracts, particularly of farnesol, citronellol, geraniol, and linalool [59,60]. Similarly, other plant extracts increased the concentration of menthol in wild mint [125] and geraniol, linalool, citronellol, and β-caryophyllene in rose-scented geranium [104].

*Phenolic compounds*

Phenolic compounds constitute one of the most important groups of plant metabolites, as they participate in a multitude of physiological processes. They can be synthesized by the shikimate/phenylpropanoid or polyketide acetate/malonate pathways [138]. In general, it has been observed that hPDBs can modulate phenolic concentrations in plant tissues. The application of higher plant-derived PHs and extracts has been found to increase phenolic compounds in different organs, such as seedlings [40], fruits [66,109,117,121], leaves [99,104,106], and roots [102,112], of multiple crops. Various types of hPDBs are reported to enhance the activity of phenylalanine ammonia-lyase (PAL), a key enzyme in the phenylpropanoid synthetic pathway, both in monocotyledons [81,87] and dicotyledons [75]. *PAL* transcription can also be promoted by hPDBs. In maize, the application of cellulosolitic dry apple hydrolysate or dry blueberry cool extract stimulated *PAL* activity and expression [83], while in tomato, an alfalfa-based PH induced the upregulation of *PAL* and *2-oxoglutarate-dependent dioxygenase* genes [123], resulting in higher levels of phenolic compounds. Furthermore, the induction of phenolic accumulation by hPDBs is also described in crops subjected to salinity [84,124], an effect which could alleviate the oxidative stress associated with this condition, given the antioxidant capacity of phenolic compounds. Conversely, hPDBs are also reported to reduce phenolic concentrations in spinach plants [105], tomato roots [112], cabbage seedlings [39], and Genovese basil subjected to Cu toxicity [55].

Flavonoids are low-molecular-weight phenolic compounds which participate in numerous physiological processes, mainly as antioxidant systems in response to stress [139], and several hPDBs are reported to increase flavonoid levels in plants [52,75,80]. For example, plants treated with hPDBs under conditions of salinity [95] or Cu toxicity [82] showed higher flavonoid levels compared to controls, indicating that the treatment reinforces plant antioxidant systems. Conversely, plants treated with hPDBs have also shown lower flavonoid levels than untreated controls [37] or those subjected to water scarcity [61]. A possible explanation is that the treated plants were healthier, with lower levels of ROS, given that the biostimulant acted to alleviate stress independently of stimulating flavonoid synthesis.

Lignin, the most abundant phenolic compound in nature, has an important structural function, so the correct regulation of its synthesis is essential for plant development. In tomato, the application of an alfalfa-based PH induced a positive regulation of *caffeoyl-CoA 3-O-methyltransferase*, a key gene in lignin biosynthesis [123].

The organoleptic quality of fruits is partly determined by their content of specialized metabolites. Treatment of habanero peppers with red grape skin extract resulted in increased levels of epicatechin, quercetin, dihydrocapsaicin, chlorogenic acid, *p*-hydroxybenzoic acid, *p*-coumaric acid, and capsaicin [65]. Likewise, a garlic extract enhanced the content of phenolic compounds in common bean pods, including protocatechuic acid, catechin, *p*-hydroxybenzoic acid, vanillic acid, and ferulic and cinnamic acid [51]. Vines treated with hPDBs produced grapes and wine with an enhanced concentration of anthocyanins in control conditions [63,64] or water scarcity [62]. In wine produced from Airén grapevines treated with vine-shoot extracts, an increase in the concentration of phenolic compounds was observed, including vanillin derivatives (vanillin and acetovanillone), volatile phenols (guaiacol and syringol), and phenolic acids (hydroxycinnamic acids) [59], whereas wine from Monastrell vines treated with oak extract showed a higher concentration of gallic acid, hydroxycinnamoyl tartaric acids, stilbenes, and the isomer piceid-*t*-resveratrol [63].

*Nitrogen-containing compounds*

N-containing compounds are mainly derived from amino acids such as phenylalanine, lysine, ornithine, tyrosine, and tryptophan [136]. Their accumulation in the plant can be modulated by the application of hPDBs, with the concentration either increasing [112,116] or decreasing [68,112]. It has been shown that under adverse conditions, the alkaloid content in plants tends to increase, as alkaloids help to alleviate stress by scavenging ROS [136]. Interestingly, although pretreatment with hPDBs is capable of inducing a higher alkaloid content in stressed plants [68,89,95], it may also result in a reduction, indicating that the biostimulant mitigates oxidative stress by an alternative pathway, obviating the need for enhanced alkaloid synthesis [55,68].

Glucosinolates are amino acid-derived specialized metabolites containing nitrogen and sulfur. Mainly found in plants of the *Brassica* genus, they play a relevant role in defense against both biotic and abiotic stresses [140,141]. Different higher plant PHs are reported to increase glucosinolates in unstressed tomato plants [112,116] and in lettuce grown under salt stress, where the upregulation of glucosinolate biosynthesis was evidenced by the accumulation of 5-(methylsulfanyl)-α-D-ribose, (E)-8-(methylsulfanyl)octanal oxime, 7-(methylsulfanyl)octanal oxime, and 7-(methylsulfanyl)heptyl-glucosinolate and the down-accumulation of their homomethionine precursors [74].

#### 4.1.3. Photosynthetic Processes

Photosynthetic metabolism includes the chemical reactions within chloroplasts, involved in the reduction in CO_2_ into carbohydrates by consuming ATP and reducing power in the form of NADPH, previously obtained through light-dependent reactions. Various biomolecules, including proteins from both photosystems and accessory pigments and ions acting as enzymatic cofactors, participate in these processes. As shown here, numerous studies have found that hPDBs can increase the components of photosynthesis, as well as improve its functioning, and more specifically, reduce or reverse the impact of abiotic stress on this vital process.

The commercial biostimulant Trainer^®^ increased the total chlorophyll content in lettuce [71,72], pea [79], spinach [106], tomato [115], and lamb’s lettuce [67], while other hPDBs have generated the same effect in maize [72,80,81], perennial wall rocket [98], cabbage [39,40], and common bean [52]. An increase in chlorophylls has also been observed after applying sorghum water extracts in camelina [41] and wheat [124] under salt stress; in the latter study, the extracts acted in synergy with benzyl aminopurine. Chlorophylls are part of light-capturing complexes, complemented by accessory pigments such as carotenoids (carotenes and xanthophylls) and anthocyanins, whose levels usually increase in parallel with those of chlorophylls after hPDB application. An increase in carotenoids was observed in treated lettuce [75], white rocket [6], rocket [103], sweet pepper [109], pea [96], maize [84], and rose-scented geranium [104]. The application of different hPDBs limited pigment loss in common bean [45,46,47,50], pea [95], maize [78], and milk thistle plants [89] under high-salinity stress. Similar results were obtained in sweet pepper plants under PHE stress [111], in squash in drought conditions [107], and in common bean plants stressed by high temperature [48].

The light absorbed by the pigments in photosystem II reaction centers is required for water photolysis, the electron transfer to the photosystems, and the electron transport chain in general. Electron transfer and photosynthetic efficiency can be quantified by measuring different parameters, many of which are improved by hPDB application. Thus, improvements were observed in photochemical efficiency (Fv/Fm ratio) in lettuce [74] and Genovese basil [55], photosystem II and photochemical efficiency in spinach [105], and net photosynthesis in olive trees [93]. In conditions of high salinity, hPDB application in Arabidopsis [37], maize [132], pea [97], okra [92], and lettuce [68] alleviated the stress impact, preserving their photosynthetic parameters, being comparable with those of control plants. In-depth studies in lettuce revealed a positive synergistic effect when hPDBs were applied with beneficial microorganisms for plant growth [117].

The reduction in carbon from CO_2_ to carbohydrates takes place in the Calvin–Benson cycle, following the generation of reducing power (NADPH) and energy molecules (ATP) in preceding light-dependent stages. Carbohydrate production requires the capture of CO_2_ through gas exchange phenomena such as transpiration and stomatal opening/closing, and specific hPDBs have been identified as enhancers of these processes. Trainer^®^ application increased net CO_2_ assimilation in tomato [117] and enhanced stomatal conductance, leaf transpiration, and net photosynthesis in cascading geranium [43]. Notably, hPDBs can either increase or maintain these parameters in plants growing under unfavorable conditions. Treatment with a cereal PH, for instance, reduced the water evaporation rate and improved drought tolerance in mung bean, rice, and wheat [91]. Under high-salinity stress, treated common bean plants maintained higher levels of relative water content, mitigating the impact of salinity [49], cowpea plants maintained high levels of net photosynthesis, transpiration rate, and relative water content [53], and eggplant plants preserved high levels of photosynthesis, transpiration rate, and stomatal conductance [54]. Maize treated with duckweed plant extract before the application of toxic concentrations of Cu preserved photosynthetic activity, evapotranspiration, stomatal conductance, and sub-stomatal CO_2_ concentration to the levels of plants grown with normal amounts of Cu [82].

In non-stressed tomato plants treated with hPDBs, metabolomics and transcriptomics studies found higher levels of vitamin K1 and 4-hydroxycoumarin, as well as an increase in transcripts such as *phytochrome interacting factor 3-like 5* (*PIL5*), *light-harvesting complex protein LHCA5*, *ferredoxin-2*, *photosystem II 22kDa protein*, *chloroplastic ATP synthase chain precursor,* and *RuBisCO*. Additionally, higher levels of metabolites and transcripts participating in light-harvesting processes, electron transport chain, and CO_2_ reduction were observed [68,123]. In zucchini plants treated with cypress leaf extract subjected to salinity stress, RuBisCO enzymatic activity was enhanced [128]. Similarly, in proteomic studies carried out in grapevine plants stressed by water deficit and treated with different PHs, an increase in proteins related to photosynthetic metabolism was observed, including RuBisCO large chain, chloroplastic ATP synthase subunit beta, photosystem II CP47 chlorophyll apoprotein, photosystem II D2 protein, chloroplastic RuBisCO activase, and chloroplastic glyceraldehyde-3-phosphate dehydrogenase A [61].

#### 4.1.4. Oxidative Metabolism

Oxidative metabolism includes all the reactions that maintain cellular redox homeostasis, defending the plant from damage caused by reactive chemical species. These molecules are present in plant physiology, being part of signaling mechanisms or being generated because of cellular metabolism. However, under stress conditions, reactive chemical species are overproduced, potentially causing damage to cellular components. Molecular markers of this damage can be quantified by analyzing lipid peroxidation, membrane stability index, and electrolyte leakage. Plant antioxidant defense responses can be enzymatic, via the activity of catalase (CAT), superoxide dismutase (SOD), peroxidase (POX), ascorbate peroxidase (APX), glutathione peroxidase (GPX), and glutathione reductase (GR), among others. Non-enzymatic defense systems include enhanced levels of osmoprotectants, hydrophilic antioxidant molecules, such as ascorbic acid, glutathione, and polyphenols, and lipophilic antioxidant molecules, such as β-carotene, lutein, and α-tocopherol. The application of hPDBs can improve antioxidant defense systems in plants by increasing the concentration of these biomolecules in stress and non-stress conditions [14,17].

Antioxidant activity, both lipophilic and hydrophilic, determined as free-radical scavenging capacity, was reported to increase after treatment with hPDBs in white rocket [6], lettuce [71], tomato [117], sweet pepper [109], radish [102], cabbage [40], rose-scented geranium [104], and lamb’s lettuce and spinach [67].

In general, hPDB mechanisms of action in crops modulate stress-related parameters, reducing electrolyte leakage and lipid peroxidation and increasing membrane stability index and the content of osmoprotectants (e.g., proline and soluble sugars) and antioxidants (e.g., ascorbic acid). Moreover, biostimulant application can enhance the transcription of genes encoding antioxidant enzymes (e.g., SOD, CAT, POX, APX, GPX, and GR) and their activities, resulting in a decrease in reactive chemical species. These results have been obtained in experiments conducted under non-stress conditions in perennial wall rocket [98], tomato [120,122,123], common bean [45], lettuce [70], and kinnow mandarin [66]. Furthermore, multiple studies have determined that the application of hPDBs increases salinity tolerance, improving the previously described parameters with respect to untreated plants and even reducing the total intracellular Na content. Such positive effects have been observed in cowpea [53], zucchini [128], milk thistle [89], pea [95,97], maize [78,84,132], camelina [41], lettuce [73], tomato [119], and common bean plants [47,48,49,50]. Similar results were obtained in common bean plants subjected to a combination of salinity and Cd contamination [46], or a single stress, such as a high temperature [48]. Regarding PHE contamination, a similar mechanism of action has been described for hPDBs applied to sweet pepper [111], potato [100], and maize plants exposed to Cu toxicity [82]; for tomato grown with limited water availability [118]; and for squash subjected to both water deficit and salinity [107].

A few studies indicate that hPDBs can also trigger a reduction in antioxidant enzyme activity compared to untreated plants, in the presence or absence of stress. This implies that the plant does not need to excessively activate the detoxifying enzymatic machinery to develop tolerance to stress, whose effects are mitigated by other detoxification mechanisms. Examples of this have been observed in treated rocket plants [103] and in maize under salt stress conditions [77,87].

#### 4.1.5. Signaling-Related Processes

Correct plant growth and development, as well as reproduction, senescence, and stress tolerance, require careful coordination of several physiological and biochemical processes. This regulation is carried out to a large extent by phytohormones that function as signaling compounds, including abscisic acid (ABA), auxins, brassinosteroids, cytokinins, ethylenes, gibberellins, jasmonates, and salicylic acid (SA) [142].

The small peptides of certain biostimulants, both PH-based and those obtained from plant extracts, are reported to act as signaling molecules, eliciting auxin- or gibberellin-like activities [81,86,105], which stimulate different metabolisms and processes, including phenolic metabolism and plant growth. However, as not all biostimulants have peptides with hormone-like properties [126], it is also important to highlight the effect of hPDBs on phytohormone accumulation patterns. For instance, the application of garlic extract in common bean was found to stimulate an increase in growth-related phytohormones, namely auxins (indole-3-acetic acid (IAA) and indole-3-pyruvate), gibberellins (gibberellin A7), and cytokinins (*trans*-Zeatin and its riboside) [51]. Positive effects of hPDBs on the accumulation of growth hormones were also observed in rocket and tomato, which had higher levels of the IAA precursors tryptamine and 4-(indol-3-yl) butanoate; the gibberellin precursor ent-7α-hydroxykaur-16-en-19-oate; and the cytokinins lupinate and *trans*-zeatin-O-glucoside-7-N-glucoside [112]. In tomato plants, the increase in growth hormones was accompanied by an upregulation of development-related genes, such as expansins, growth-regulating factors 3 (*GRF3*) and 5 (*GRF5*), and *lob domain protein 1* [123]. However, certain biostimulant treatments may result in a lower concentration of these hormones and their precursors, as evidenced by a reduction in brassinosteroid levels in tomato [112,116].

Phytohormones have a role in mediating stress response mechanisms, even presenting a crosstalk in regulating both abiotic and biotic stress-induced signaling. ABA is a major phytohormone in mediating these processes, and its role as a master regulator stands out. In this sense, the effect of hPDBs on the phytohormone profile has also been investigated in plants growing under unfavorable conditions [143]. Pretreatment with moringa leaf extract of Arabidopsis plants grown under saline conditions enhanced the transcription of the *AtIAA*, *ABI5,* and *PR1* genes, related to the auxin, ABA, and SA synthetic pathways, respectively [38]. The application of different PHs in Arabidopsis induced the accumulation of the brassinosteroid precursors 6-deoxo-24-epicathasterone and campest-5-en-3-one and a reduction in derivative forms of plant hormones such as benzyladenine-7-glucoside, 16,17-dihydro-16α-17-dihydroxy gibberellin 12, and methylgibberellin 4; the IAA-derivate 4-(indol-3-yl)butanoyl-β-D-glucose; or the brassinosteroid castasterone compared to untreated plants [37]. The decrease in degraded or inactivated hormone products could signify a preservation of homeostasis regulated by the active hormone, whereas lower castasterone levels could indicate an amelioration of the effects of salt toxicity [37]. The preservation of auxin, cytokinin, gibberellin, brassinosteroid, and jasmonate levels under stress conditions by hPDBs has also been described in lettuce [69] and common bean subjected to salinity [49] and heat [48], maize exposed to Cu toxicity [82], and grapevines grown under water scarcity [61]. However, a negative modulation was noted in lettuce under salt stress [68] and tomato subjected to water scarcity [118].

The modulation of phytohormone levels can activate signaling cascades that regulate various processes, including stress response. A transcriptomic study in tomato plants proposed the induction of a signaling cascade by an alfalfa-based PH. Thus, this biostimulant originated an upregulation of genes involved in hormone synthesis, causing the activation of a phosphorylation cascade involving different protein kinases (e.g., CPK9, CPK28, CRCK3, LRR kinases, MAPKKK21, Pi kinase, PEPKR2, and WAK2), which regulate transcription factors (e.g., bHLH, APETALA2, homeobox-leucine zipper, PPR, WRKY, Myb, ERF2, and Rav). Together, these cascades would lead to the expression of genes encoding stress response proteins (ABC transporters, DC1 domain-containing proteins, alternative oxidase 1A, cytochrome 450, leucine-rich repeat proteins, heat shock proteins, L-threonine ammonia-lyase, aldo/keto reductase, threonine ammonia-lyase and chitinases, glutathione-S-transferases, and wound-induced proteins, among others) [123].

### 4.2. Whole-Plant Level

In addition to impacting cellular and molecular processes, biostimulants exert effects at the whole-plant level, influencing physiological processes such as germination, root and shoot growth and morphology, flowering, and fructification. From the agronomic perspective, these changes are highly important, as they are closely linked to crop yield (defined as the mass or volume of plant biomass per unit of cultivated land) and quality, thus determining economic profitability [144].

#### 4.2.1. Germination

Germination is one of the most critical processes in agriculture, as it determines plant growth and development, as well as the subsequent crop yield and fruit quality. Therefore, it is essential to develop strategies that ensure a successful and homogeneous germination, even in plants commencing their life cycle under stress conditions [144]. The application of hPDBs has proven effective in promoting germination. For example, higher plant-derived PHs increased the germination percentage in mung bean and wheat [91]. Furthermore, studies performed in maize report that treatment with Trainer^®^ increased the length of the coleoptile, a distinctive part of grass embryos, showing a similar effect to IAA. This result suggests that Trainer^®^ could have an auxin-like function in the germination process [79].

Unfavorable conditions negatively affect the germination process, decreasing the total number of germinated seedlings and their size. hPDBs are also capable of counteracting the negative impact of abiotic stresses on germination. Several higher plant-derived PHs were found to maintain higher survival rates in primed Arabidopsis seeds subjected to high salinity, also ensuring a more homogeneous germination [37]. This beneficial effect, as well as better growth rates of seedlings, has also been observed under salinity in crops of agronomic interest such as zucchini [128], pea [95,97], camelina [41], wheat [124], and maize [78,132].

#### 4.2.2. Root Growth and Morphology

Plant growth and development depend strongly on root absorption of nutrients and water, which is essential for achieving high biomass production and successful harvests [145]. Various hPDBs have shown the ability to promote rooting, resulting in increased root dry weight, length, area, and number in dicotyledons such as cabbage [39,40] and tomato [79,112,115,116,120,122] and monocotyledons such as maize [80,81,83]. This effect has also been verified in the soilless cultivation of lily plants, in which the application of alfalfa-derived biostimulants generated a longer root system compared to untreated plants [76].

The effective absorption of nutrients depends on the formation of adventitious roots and root hairs. In field mustard, bioactive peptides present in hydrolysates derived from soybean by-products not only increased root thickness, but also generated a higher number of adventitious roots and root hairs of greater length. In addition, an increase in trichoblasts and atrichoblasts was observed, although their location pattern did not change, which indicates that the biostimulant promoted root growth differently from ethylene [126]. In cannabis plants, treatment with a biostimulant formulation composed of aloe vera, fish, and kelp extracts increased the number of root tips and branch points, as well as the total surface area of the root system [42].

In plant species where the root is the edible part, promoting root growth improves crop profitability. The application of various leaf aqueous extracts in radishes increased root length, therefore resulting in an improved harvest yield. Moreover, the treated radishes had an improved content of bioactive compounds and greater antioxidant capacity [102].

The application of biostimulants may also protect roots from abiotic stresses, ensuring their optimal functionality. Tested in conditions of salinity, biostimulants promoted better root growth and morphology in maize [77,84], onion [94], camelina [41], common bean [47], and lettuce [74], as well as in maize subjected to Cu toxicity [82]. Under stress conditions associated with alkaline substrates, the application of Trainer^®^ increased the effectiveness of microbial biostimulants in a synergistic process, improving lettuce root growth [70]. After treatment with moringa leaf extract, common bean plants subjected to high temperatures also showed a better response to stress than untreated plants, producing longer roots and more biomass [48].

#### 4.2.3. Shoot Growth and Morphology

Often, shoot growth parameters are indicative of the future yield of the crop. As well as increasing plant height and stem diameter, the action of different biostimulants can positively modulate shoot dry weight and length. For example, Trainer^®^ is reported to increase plant height and shoot dry biomass in maize [72], shoot dry weight and length in tomato and dwarf pea [79,115,121], and plant height of cascading geranium [43]. Biostimulants derived from plant by-products generated a similar response, exemplified by carob germ-based extracts, which increased stem diameter and plant height in tomato [122,127].

Regarding vascular tissues, cowpea plants treated with ammi seed extract showed larger leaf blades, more palisade and spongy tissue, and thicker phloem and xylem than the untreated controls, as well as a larger stem and vessel diameters and more vascular bundles [53].

The leaf area, a determining factor in photosynthetic capacity, can also be increased by biostimulants, as reported in cascading geranium [43], tomato [114,120,122], and maize [86]. In maize and olive trees, the application of hPDBs of different origins increased leaf biomass, another parameter of plant growth [80,81,83,93].

In the floriculture crop gladiolus, moringa leaf extracts increased plant height, corm biomass and diameter, and cormel production [56,57]. Similarly, in lily plants, the application of an alfalfa-based PH enhanced bulb size and resulted in greener and more expanded blades [76].

In leafy crops where the marketable/consumable part is mainly the shoot, such as cabbage, rocket, lettuce, or spinach, the positive effect of biostimulants on growth is of special interest as their action can increase crop yield. hPDBs are reported to increase the height and biomass of cabbage [39,40], rocket [103], perennial wall rocket [98,99], and white rocket shoots [6], increasing their total yield. In different types of lettuce and lamb’s lettuce, the application of biostimulants also promoted growth and a higher fresh yield [67,71,75], an effect also observed in Genovese basil [55] and spinach, which also showed a greater leaf area [67,105,106]. In wild mint, large-leaf beauty berry extracts enhanced growth and leaf area and number [125], while in rose-scented geranium, moringa leaf extracts increased plant height, branch number, and leaf area, also enhancing overall yield and volatile oil content [104]. In addition, horticultural products had an improved nutritional quality, due to a higher concentration of minerals, sugars, and phenols and antioxidant capacity, as well as an increased concentration of chlorophylls, as mentioned above, which gives a better quality to the horticultural product [6,98,106].

Biostimulant treatments can also avoid the negative impact of abiotic stresses on plant size. Thus, under saline conditions, several hPDBs are reported to increase the height and biomass in maize [77,84], camelina [41], tomato [68], lettuce [68,69,70,73,74], mung bean [90], and milk thistle shoots [89]; the height and leaf biomass in onion plants [94]; the height, biomass, leaf blade size, phloem and xylem thickness, vessel diameter, and number of vascular bundles in cowpea [53]; and plant height and leaf elongation and expansion in wheat [124] and common bean, where the number of leaves and leaf area also increased [45,47,49,50]. hPDB effectiveness has also been shown under other unfavorable conditions. In tomato and squash plants, the application of Trainer^®^ attenuated the negative effect of water scarcity, increasing the biomass of treated plants [107,118]. Similarly, duckweed extract promoted shoot growth in maize plants subjected to Cu toxicity [82]. Under PHE stress, moringa seed extract increased tuber yield and reduced PHE accumulation in potato [100]. Lastly, common bean plants subjected to heat stress produced longer shoots and more biomass after treatment with moringa extract [48].

#### 4.2.4. Flowering

The effect of hPDBs on flowering is of great interest, not only because this process is closely linked to fruit production, but also because it is the main determinant of quality in floriculture crops and ornamental plants. Studies have found that biostimulants are capable of promoting flowering, increasing the number of flowers in various crops, from food crops such as common bean [51] and ornamental plants such as cascading geranium [43]. As well as increasing the number of flowers produced per plant [43,127] and the diameter of the flower buds, biostimulants can also shorten the crop cycle, thereby advancing the flowering process [76]. Treatment of gladiolus with moringa leaf extract not only resulted in earlier spike emergence and an increase in the number of spike florets per plant, but also extended its maximum vase life in sucrose [57]. Thus, hPDBs offer a broad spectrum of benefits for the cultivation of ornamental crops, ranging from accelerated flowering to improvements in flower quantity, size, and post-harvest characteristics.

#### 4.2.5. Fructification and Fruit Quality

Among the myriad of actions that biostimulants perform at different stages of plant development, their role in boosting fructification is of particular economic importance. Their application can increase the weight, size, and number of fruits produced per plant [101,127] as well as improve overall fruit quality. In tomato plants, biostimulant treatments increased fruit production and enhanced the concentration of minerals (mainly K and Mg), bioactive compounds such as lycopene, phenols, ascorbic acid, organic acids (malate, oxalate, citrate, and isocitrate), and soluble solids. The treated tomatoes also had greater antioxidant activity and fruit brightness and redness [113,114,117,121].

Regarding improvements in fruit quality, the application of plant extracts resulted in a lower incidence of cracking in sweet cherries due to enhanced fruit firmness and Ca concentration. As well as a higher fruit production, a greater proportion of cherries were of the color preferred by consumers [108]. Similarly, treatment of kinnow mandarin with moringa leaf extracts led to a higher fruit yield, a reduction in fruit drop, and a higher content of fruit sugars, vitamin C, antioxidants, and phenols [66]. In mango fruits, a combination of roselle and garlic extracts generated a far greater response than the sum of individual effects when applied separately, improving fruit set, retention, yield, and quality, in addition to its nutritional content (mainly increased levels of N, P, and K) [88]. Likewise, the synergy of an alfalfa-based PH and red grape extracts applied to habanero pepper plants increased fruit weight and number and enhanced their bioactive properties due to a higher content of glucose and specialized metabolites [65]. Biostimulants also had a positive effect on sweet pepper plants, increasing total fruit yield, vitamin C, total phenolic content, and antioxidant activity [109,110].

In winemaking, the organoleptic properties and overall quality of wine are closely linked to the primary and specialized metabolite contents of grapes. In an innovative and sustainable approach involving the revalorization of a waste by-product, extracts from vine-shoot residues have been applied as biostimulants and found to modify the phenolic composition, mineral content, anthocyanin concentration, volatile and glycosylated compounds, and color characteristics of grapes [58,59,60,63,64].

An investigation into the effects of biostimulants on legumes is of considerable importance, given their widespread consumption in many regions [146]. The application of different higher plant extracts in common bean improved crop yield, increasing pod number, pod fresh weight, and seed yield [45,49,51,52]. Similar effects were also observed in pea plants treated with moringa extracts, with an increase in seed weight and contents of protein and minerals (especially K, Ca, Mg, Fe, and P) [96].

A primary consequence of stress is a negative impact on fruit production, leading to a reduction in both quantity and quality. Biostimulants have proven effective in mitigating these adverse effects, as described in numerous studies. Under conditions of water scarcity, biostimulants positively influenced vine crop yields and enhanced the anthocyanin content of grapes [62]. In legumes subjected to saline conditions, including common bean, mung bean, cowpea, and okra, different plant extracts have been successfully used to improve crop yield, increasing the number of pods and seeds per pod [46,47,50,53,90,92]. A protective effect of biostimulants against salinity has also been demonstrated in solanaceous plants such as eggplant and sweet pepper, with the treated plants producing more fruits of higher weight [54,111]. A similar positive impact was observed in sweet pepper and common bean subjected to PHE stress [46,111].

**Figure 3:**
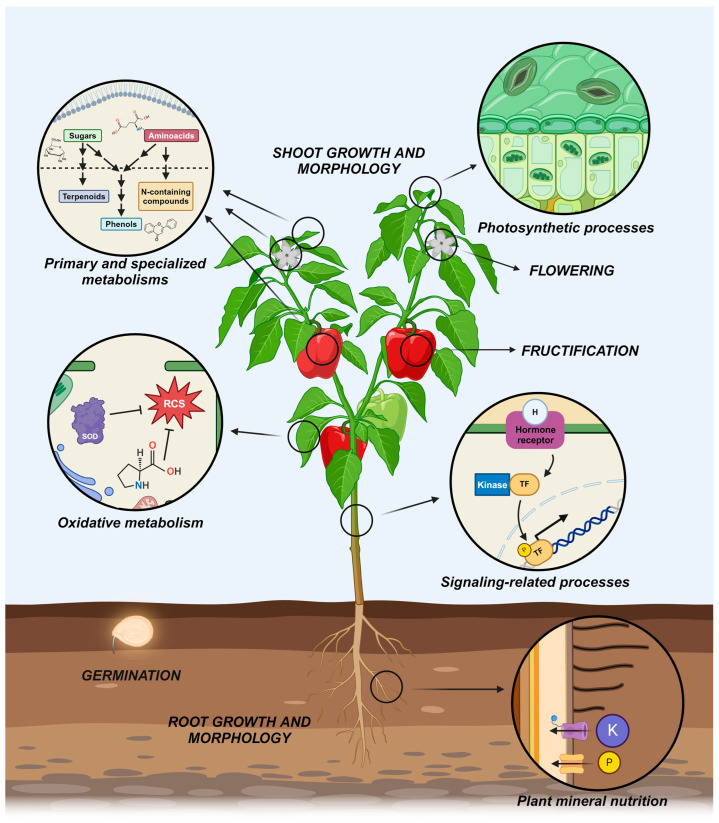
Schematic representation of the different metabolisms and processes affected by hPDBs in plants. Created with BioRender.com, accessed on 31 January 2024.

All the different metabolisms and processes affected by hPDB-induced mechanisms of action are summarized in Figure 3.

## 5. Conclusions and Future Perspectives

The application of biostimulants constitutes an alternative approach toward a more sustainable agriculture that is less reliant on fertilizers and agrochemicals. Those derived from natural sources are of particular interest, especially hPDBs, given their ecological advantages and broad spectrum of action on plant physiology. The ability of these biostimulants to modulate cellular reactions and physiological processes can alleviate the effects of abiotic stress on plants growing under unfavorable edaphoclimatic conditions. Understanding biostimulant mechanisms of action underlying these effects, and ultimately the modes of action, is vital for the design and customization of biostimulant formulations for each type of crop. With this aim, omics tools offer the possibility of elucidating the composition of the biomolecules in each formulation, as well as the modifications they induce in plant physiology. Simultaneously, it is also necessary to study different methods of biostimulant application, exploring key aspects such as the duration of the effect, optimal application frequency, the stage of the plant life cycle when application is most effective, and how biostimulants may interfere with the action of fertilizers and agrochemicals. Then, new sources of hPDBs should be uncovered to facilitate their large-scale industrial production.

## Figures and Tables

**Table 1:** Main hPDBs used in crops and their application method.

Crop	hPDB	Application Method	Ref.
Arabidopsis (*Arabidopsis thaliana*)	Moringa (*Moringa oleifera*) leaf extract, Trainer^®^, Vegamin^®^, and PHs from Fabaceae, Malvaceae, Brassicaceae, Solanaceae, and Graminaceae	Seed priming	[37,38]
Cabbage (*Brassica oleracea*)	Extracts from mugwort (*Artemisia vulgaris*), calendula (*Calendula officinalis*) flowers, purple coneflower (*Echinacea purpurea*) flowers and leaves, chamomile (*Matricaria chamomilla*) flowers, basil (*Ocimum basilicum*), giant goldenrod (*Solidago gigantea*) leaves, comfrey (*Symphytum officinale*) root, dandelion (*Taraxacum officinale*) flowers, leaves, and root, valerian (*Valeriana officinalis*) root, aloe vera (*Aloe vera*) leaves, chokeberry (*Aronia melanocarpa*) fruits, red beet (*Beta vulgaris*) root, horsetail (*Equisetum arvense*), common sea buckthorn (*Hippophae rhamnoides*) fruits, hypericum (*Hypericum perforatum*), red lentil (*Lens culinaris*) seeds, common bracken (*Pteridium aquilinum*) leaves, knotgrass (*Polygonum aviculare*), pea (*Pisum sativum*) seeds, broadleaf plantain (*Plantago major*), red clover (*Trifolium pratense*) flowers, and nettle (*Urtica dioica*) leaves and root	Foliar spray	[39,40]
Camelina (*Camelina sativa*)	Shorgum (*Shorgum* sp.) water extract	Seed priming	[41]
Cannabis (*Cannabis sativa*)	Aloe vera (*Aloe vera*) extract and aloe vera, fish, and kelp complex	Nutrient solution	[42]
Cascading geranium (*Pelargonium peltatum*)	Trainer^®^	Foliar spray	[43]
Common bean (*Phaseolus vulgaris*)	Extracts from fish bean (*Tephrosia vogelii*) and tree marigold (*Tithonia diversifolia*), garlic (*Allium sativum*) cloves, licorice (*Glycirrhiza glabra*) root, moringa (*Moringa oleifera*) leaves, and tomato (*Solanum lycopersicum*) powder hydrolysate.	Foliar spray, seed priming, and substrate application	[44,45,46,47,48,49,50,51,52]
Cowpea (*Vigna unguiculata*)	Extracts from fennel (*Foeniculum vulgare*) and ammi (*Ammi visnaga*) seeds	Foliar spray	[53]
Eggplant (*Solanum melongena*)	Sugarbeet (*Beta vulgaris*) extract	Foliar spray	[54]
Genovese basil (*Ocimum basilicum*)	Trainer^®^	Foliar spray	[55]
Gladiolus (*Gladiolus grandiflorus*)	Extracts from moringa (*Moringa oleifera*) leaves, garlic (*Allium sativum*) extract, and licorice (*Glycyrrhiza glabra*) root	Foliar spray	[56,57]
Grapevine (*Vitis vinifera*)	Trainer^®^, Stimtide^®^, and soybean (*Glycine max*) hydrolysate and extracts from french oak (*Quercus sessiliflora*), vine-shoot (*Vitis vinifera*) waste, and carob germ (*Ceratonia silique*) and from mixture of maize (*Zea mays*) and sorghum (*Sorghum* sp.) distiller’s dried grains.	Foliar spray and substrate application	[58,59,60,61,62,63,64]
Habanero pepper (*Capsicum chinensis*)	Red grape (*Vitis vinifera*) skin extract and alfafa (*Medicago sativa*) hydrolysate	Foliar spray	[65]
Kinnow mandarin (*Citrus nobilis* × *Citrus deliciosa*)	Moringa (*Moringa oleifera*) leaf extract	Foliar spray	[66]
Lamb’s lettuce (*Valerianella locusta*)	Trainer^®^	Foliar spray	[67]
Lettuce (*Lactuca sativa*)	Auxym^®^, LISIVEG^®^, Trainer^®^, Vegamin^®^, and PHs from Fabaceae, Malvaceae, Brassicaceae, Solanaceae, and Graminaceae. Extracts from moringa (*Moringa oleifera*) leaves and leaves and flowers of borage (*Borago officinalis*).	Foliar spray, seed priming, root application, and substrate application before transplant.	[68,69,70,71,72,73,74,75]
Lilium ‘Brindisi’ (*Lilium longiflorum* × *Lilium x elegans*)	Alfalfa (*Medicago sativa*) hydrolysate	Foliar spray and substrate application	[76]
Maize (*Zea mays*)	Lignin nanoparticles from olive (*Olea europaea*) waste, Trainer^®^, hydrolysates from alfalfa (*Medicago sativa*), dry apple (*Malus domestica*), and extracts from carrot (*Daucus carota*) root, blueberry (*Vaccinium corymbosum*) fruits, duckweed (*Lemna minor*), hawthorn (*Crataegus monogina*) leaves, common grapevine (*Vitis vinifera*) grape skin, rosemary (*Rosmarinus officinalis*), white wormwood (*Artemisia herba-alba*), whortleberry (*Vaccinium arctostaphylos*) fruit, and willow tree (*Salix babylonica*) barks and leaves	Foliar spray, nutrient solution, seed priming, and substrate application	[72,77,78,79,80,81,82,83,84,85,86,87]
Mango (*Mangifera indica*)	Roselle (*Hibischus sabdariffa*), garlic (*Allium sativum*) clove, and algae extracts alone or combinated	Foliar spray	[88]
Milk thistle (*Silybum marianum*)	Moringa (*Moringa oleifera*) leaf extract	Substrate application	[89]
Mung bean (*Vigna radiata*)	Cereal PHs and angosteen (*Garcinia mangostana*) pericarp extract	Foliar spray and seed priming	[90,91]
Okra (*Abelmoschus esculentus*)	Sugarbeet (*Beta vulgaris*) extract	Foliar spray	[92]
Olive (*Olea europaea*) tree	Duckweed (*Lemna minor*) plant extract	Foliar spray	[93]
Onion (*Allium cepa*)	Mimosa (*Acacia dealbata*) bark extract	Foliar spray	[94]
Pea (*Pisum sativum*)	Trainer^®^ and extracts from licorice (*Glycyrrhiza glabra*) root, moringa (*Moringa oleifera*) leaves, and narrow-leaf cattail (*Typha angustifolia*) leaves	Foliar spray, seed priming, and shoot application	[79,95,96,97]
Perennial wall rocket (*Diplotaxis teniufolia*)	Auxym^®^, Trainer^®^, and their combination	Foliar spray	[98,99]
Potato (*Solanum tuberosum*)	Moringa (*Moringa oleifera*) seed extract	Substrate application	[100]
Pulasan (*Nephelium ramboutan-ake*)	ComCat^®^	Foliar spray	[101]
Radish (*Raphanus sativus*)	Extracts from leaves of mulberry (*Morus nigra*), brassica (*Brassica napus*), sorghum (*Sorghum bicolor*), and moringa (*Moringa oleifera*)	Foliar spray	[102]
Rice (*Oryza sativa*)	Cereal PHs	Foliar spray	[91]
Rocket (*Eruca vesicaria*)	Moringa (*Moringa oleifera*) leaf and twig extracts	Foliar spray	[103]
Rose-scented geranium (*Pelargonium graveolens*)	Moringa (*Moringa oleifera*) leaf extract	Foliar spray	[104]
Spinach (*Spinacia oleracea*)	Amalgerol^®^ and Trainer^®^	Foliar spray	[67,105,106]
Squash (*Curcubita pepo*)	Moringa (*Moringa oleifera*) leaf extract	Foliar spray	[107]
Sweet cherry (*Prunus avium*)	Auxym^®^	Foliar spray	[108]
Sweet pepper (*Capsicum annuum*)	Radifarm^®^, Megafol^®^, Viva^®^, and Benefit^®^ and extracts from moringa (*Moringa oleifera*) seeds and leaves and licorice (*Glycyrrhiza glabra*) root	Foliar spray, nutrient solution, and substrate application	[109,110,111]
Tomato (*Solanum lycopersicum*)	Auxym^®^, Trainer^®^, Vegamin^®^, PHs from alfalfa (*Medicago sativa*), Fabaceae, Malvaceae, Brassicaceae, Solanaceae, and Graminaceae and extracts from crushed maize (*Zea mays*) grain, garlic (*Allium sativum*) cloves, seagrass (*Zostera marina*), barley (*Hordeum vulgare*), and grain waste and processing residues from fennel (*Foeniculum vulgare*) and lemon (*Citrus limon*)	Foliar spray, seed priming, nutrient solution, cutting immersion, and substrate application	[68,79,112,113,114,115,116,117,118,119,120,121,122,123]
Wheat (*Triticum aestivum*)	Cereal PHs and shorgum (*Shorgum* sp.) water extract	Foliar spray and seed priming	[91,124]
White rocket (*Diplotaxis erucoides*)	Auxym^®^ and Trainer^®^	Foliar spray	[6]
Wild mint (*Mentha arvensis*)	Calliterpenone from large-leaf beauty berry (*Callicarpa macrophylla*) extract	Sucker immersion	[125]
Wild mustard (*Brassica rapa*)	Soybean (*Glycine max*) waste hydrolysate	Root application	[126]
Wild tomato (*Solanum pimpinellifolium*)	Carob (*Ceratonia siliqua*) germ hydrolysate extract	Substrate application	[127]
Zucchini (*Curcubita pepo*)	Cypress (*Cupressus macrocarpa*) leaf extract	Seed priming	[128]
